# Visualization of microaneurysms in macular telangiectasia type 1 on optical coherence tomography angiography before and after photocoagulation

**DOI:** 10.1007/s00417-020-04953-9

**Published:** 2020-10-02

**Authors:** Mitsuko Nakai, Hisashi Iwami, Hisashi Fukuyama, Fumi Gomi

**Affiliations:** Department of Ophthalmology, Hyogo College of Medicine, 1-1, Mukogawa, Nishinomiya, 663-8501 Japan

**Keywords:** Macular telangiectasia, Optical coherence tomography angiography, Microaneurysms, Photocoagulation

## Abstract

**Purpose:**

To evaluate changes in the visualization of microaneurysms (MAs) in cases of macular telangiectasia (Mac Tel) type 1 on optical coherence tomography angiography (OCTA) before and after treatment with direct photocoagulation and to evaluate their relationship with treatment efficacy.

**Methods:**

The study included 12 eyes from 12 patients (8 men, 4 women; mean age 72.1 years) with Mac Tel type 1 accompanied by cystoid macular edema. OCTA for the evaluation of MAs was performed before and 15 min and 6, 12, and 24 weeks after photocoagulation. The best-corrected visual acuity (BCVA) and central retinal thickness (CRT) were also evaluated.

**Results:**

A total of 73 MAs were detected within the areas of macular edema on OCTA, and 39 of these underwent photocoagulation. At 15 min after treatment, 17 MAs were no longer visible on OCTA. At 6 weeks, two MAs had reappeared, whereas five additional MAs were no longer visible. The CRT in eyes with resolved MA was significantly less than that in eyes with persistent MAs (*p* = 0.016). At 24 weeks, seven eyes had no visible MAs, and the BCVA was not significantly different from baseline.

**Conclusion:**

OCTA can monitor changes in the visualization of MAs associated with Mac Tel type 1 after direct photocoagulation. Eyes in which MAs disappeared after treatment could recover from cystoid macular edema.

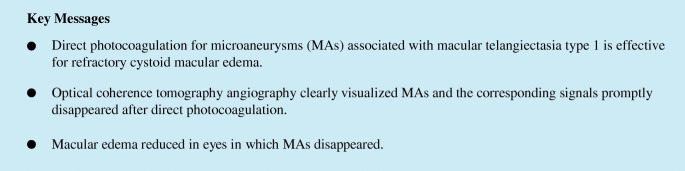



## Introduction

Macular telangiectasia (Mac Tel) is the name for a group of rare diseases that cause spontaneous capillary dilatation within the macula. This condition was first described by Gass and Oyakawa in 1982 as idiopathic juxtafoveal retinal telangiectasia [1]. Yannuzzi et al. later referred to it as idiopathic macular telangiectasia and classified into three types on the basis of optical coherence tomography (OCT) findings [2]. In Japan, Mac Tel type 1, which is characterized by aneurysmal telangiectasia, has a higher prevalence than Mac Tel type 2 [3]. Mac Tel type 1 is more often found in men, is usually unilateral, and is characterized by exudative lesions due to telangiectasia on the temporal side of the macula.

Mac Tel type 1 is often encountered in clinical practice, because cystoid macular edema (CME) associated with focal capillary dilatation and microaneurysms (MAs) causes visual deterioration [1, 2]. Currently, fluorescein angiography (FA) is necessary to assess the location of lesions in telangiectasia and the severity of leakage. Indocyanine green angiography (ICGA) is also useful in depicting the size, location, and morphology of each lesion, because diffusion of ICGA through the small fenestrations of retinal vessels is limited owing to its protein-bonding nature within the blood [4, 5]. However, FA and ICGA are invasive and time-consuming.

Optical coherence tomography angiography (OCTA) is a recently established technology to visualize retinal-choroidal vessels using OCT principles; it can be performed repeatedly because of its noninvasive nature. OCTA provides more detailed information on retinal vascular abnormalities than OCT [6–10].

To date, there is no proven therapy for Mac Tel; however, anti-vascular endothelial growth factor (VEGF) drugs are sometimes administered to resolve CME, but the improvement is reported to be transient [11]. As in cases of MAs associated with diabetic maculopathy, the basic treatment for persistent CME in Mac Tel type 1 is direct application of photocoagulation to abnormally dilated retinal capillary lesions including MAs [12, 13]. To increase the success rate, navigated laser photocoagulation using FA [14] and ICGA [4, 5] is suggested. However, as an alternative to the use of these invasive forms of angiography, OCTA-guided photocoagulation can be helpful because it can demonstrate prompt changes in blood flow. In this study, we evaluated short-term changes in the visualization of MAs on OCTA in cases of Mac Tel type 1 before and after treatment with focal photocoagulation and further evaluated their relationship with treatment efficacy.

## Materials and methods

This retrospective study enrolled consecutive patients with CME due to Mac Tel type 1 who were diagnosed and treated with photocoagulation from June 2016 to December 2019 at Hyogo College of Medicine. The Institutional Review Board of Hyogo College of Medicine approved this study (No. 2426), which followed the tenets of the Declaration of Helsinki. Informed consent was obtained from all subjects.

Patients were comprehensively diagnosed with Mac Tel type 1 according to their medical history, FA, and ICGA findings, and the condition of the fellow eye. Patients with neovascular maculopathies (e.g., age-related macular degeneration, polypoidal choroidal vasculopathy, and retinal angiomatous proliferation) or other retinal vascular diseases such as diabetic maculopathy and retinal vein occlusion, were excluded.

The treatment criteria for direct photocoagulation were that MAs (1) were visible on FA, ICGA, and OCTA, (2) visible leakage on FA associated with persistent or refractory CME involving the fovea, (3) had a size of 50 μm or more (larger than the size of the laser spot, to avoid damage to surrounding tissue), and (4) did not show involvement within the foveal avascular zone. After pupillary dilation and instillation of topical anesthesia, photocoagulation at a wavelength of 577 nm (NIDEK, MC-500, Tokyo, Japan) was performed by two retina specialists (FG and HI). The laser parameters were as follows: (1) spot size of 50–100 μm; (2) pulse duration of 0.05–0.1 s; (3) single spot; and (4) burn intensity of 80–140 mW, so that only the lesions of the MAs became gray or white. A combined anti-VEGF drug injection was administered at the discretion of the physician.

All patients underwent ophthalmological examinations including best-corrected visual acuity (BCVA), fundus examination, OCT, and OCTA (RS-3000, NIDEK, Japan, or DRI OCT Triton, Topcon, Japan) before and after treatment. The OCTA examination used a 3.0 × 3.0 or 4.5 × 4.5-mm square area and was performed before and just after photocoagulation (within 15 min), and around 6, 12, and 24 weeks thereafter. The same OCTA device was used repeatedly for the examination of each given patient, and default segmentation slabs of the superficial and deep retina were used in each device. The number and visibility of MAs in each slab of the superficial and deep retina on enface OCTA within the 3-mm field of the Early Treatment of Diabetic Retinopathy Study (ETDRS) circle were independently evaluated for each time point by two examiners (MN and HI), without reference to FA or ICGA. If the interpretations differed, a third observer (FG) interpreted the results. Then, the number of MAs on the baseline OCTA was compared with that at the corresponding area on FA or ICGA. Additional treatment was administered between 6 and 24 weeks, according to the physician’s decision.

The decimal BCVA, obtained using a Landolt C chart, was converted to a logarithm of minimum angle resolution (logMAR). The changes in BCVA and central retina thickness (CRT) were also compared before and after the treatment.

## Statistical analysis

All statistical analyses were performed using EZR software (Saitama Medical Center, Jichi Medical University). Continuous variables are expressed as the mean ± standard deviation (SD). The Wilcoxon signed-rank test and Friedman test were used to compare the microaneurysm lesions, BCVA, and CRT before and after photocoagulation. *P* values of < 0.05 were considered statistically significant.

## Results

A total of 12 eyes in 12 patients (8 men and 4 women) were included in the study (Table 1). The mean age of the patients was 72.1 ± 8.4 years (range, 56–81 years). One or more lesions of telangiectasia and MAs were detected on FA and OCTA. ICGA was obtained in 11 patients. Nine eyes (75%) had a history of treatment with anti-VEGF drugs and/or sub-Tenon’s triamcinolone acetonide injection within 3 months, but CME remained.

**Table 1:** Patient characteristics and treatment outcomes

Case	Sex	Age (years)	Prior treatment within 3 months	Combined anti-VEGF	Additional treatment		BCVA (logMAR)				CRT (μm)			
					6–12 weeks	12–24 weeks	baseline	6 weeks	12 weeks	24 weeks	baseline	6 weeks	12 weeks	24 weeks
1	M	56	STTA	–	–	PC	0.16	0.16	0.22	0.22	389	335	317	295
2	F	80	STTA, Anti-VEGF	–	PC	–	0.22	− 0.08	0.10	0.10	354	333	304	288
3	M	79	STTA, Anti-VEGF	–	PC	PC Anti-VEGF	0.16	0.16	0.22	0.22	350	372	420	309
4	F	75	–	–	PC	PC	0.3	0.05	0.05	0	524	530	431	294
5	M	81	STTA	–	–	–	0.22	0.16	0.16	0.30	441	385	360	356
6	F	81	Anti-VEGF	–	–	–	0.16	0.16	0.16	0.16	384	318	304	304
7	M	76	Anti-VEGF	+	PC Anti-VEGF	–	0.30	0.22	0.22	0.05	393	330	334	307
8	F	72	Anti-VEGF	+	–	–	0	0	0	0	383	212	215	254
9	M	74	Anti-VEGF	–	PC Anti-VEGF	PC	0.30	0.30	0.30	0.40	371	375	316	491
10	M	57	–	–	–	–	− 0.08	− 0.08	− 0.08	− 0.08	333	262	262	259
11	M	70	STTA, Anti-VEGF	–	–	–	0.22	0.30	0.30	0.30	432	325	324	289
12	M	64	–	+	–	–	0.1	0	N.A.	0	464	304	N.A.	302

*BCVA*, best-corrected visual acuity; *logMAR*, logarithm of the minimum angle of resolution; *CRT*, central retinal thickness; *VEGF*, vascular endothelial growth factor; *STTA*, sub-Tenon’s triamcinolone acetonide injection; *PC*, photocoagulation

At baseline, the mean logMAR BCVA was 0.17 ± 0.11 (range, − 0.08 to 0.30). All patients complained of blurred vision and metamorphopsia associated with the CME. The CRT values averaged 401.50 ± 52.29 μm (range, 333–524 μm).

The numbers of MAs on FA and OCTA are shown in Table 2. On OCTA, microvascular abnormalities consisting of tortuous capillaries and MAs were found in the superficial and deep retinal slabs in all eyes. A total of 73 MAs were visualized on OCTA; 65 MAs in the deep retinal slab and 25 MAs in the superficial retinal slab. Seventeen relatively large MAs were seen in both superficial and deep retinal layers, whereas eight MAs were only seen in the superficial layer. Compared with the images on FA and ICGA, the visibility of MAs on OCTA was inferior, and some MAs were not detectable. However, some hyperreflective dots seen on OCTA were derived from hard exudates.

**Table 2:** Visualization of microaneurysms on optical coherence tomography angiography

Case	MAs on FA (*n*)	MAs on baseline OCTA (*n*)			MAs on OCTA at 15 min (*n*)			MAs on OCTA at 6 weeks (*n*)			MAs on OCTA at 12 weeks (*n*)			OCTA at 24 weeks, visible MAs (*n*)		
		*S*	*D*	Both	*S*	*D*	Both	*S*	*D*	Both	*S*	*D*	Both	*S*	*D*	Both
1	17	2	13	2	1	7	1	1	8	1	1	8	1	1	8	1
2	4	1	2	1	0	1	0	0	2 (new 1)	0	0	2 (new 1)	0	0	2 (new 1)	0
3	3	0	2	0	0	2	0	0	2	0	0	2	0	0	0	0
4	30	12	13	5	5	8	2	1	5	1	1	5	1	1	5	1
5	2	1	3	1	0	1	0	0	1	0	0	0	0	0	0	0
6	1	1	4	1	0	3	0	0	3	0	0	3	0	0	3	0
7	4	0	4	0	N.A.	N.A.	N.A.	0	2	0	0	2	0	0	0	0
8	3	0	2	0	0	1	0	0	0	0	0	0	0	0	0	0
9	8	0	5	0	0	3	0	0	3 (new 1)	0	0	3 (new 1)	0	2 (new 2)	3 (new 2)	2
10	1	0	1	0	0	1	0	0	0	0	0	0	0	0	0	0
11	2	2	5	2	0	3	0	0	0	0	0	0	0	0	0	0
12	21	6	11	5	5	10	5	4	9	4	N.A.	N.A.	N.A.	4 (new 1)	10 (new 3)	4

*MAs*, microaneurysms; *OCTA,* optical coherence tomography angiography; *S*, superficial retinal slab; *D*, deep retinal slab; *N.A.*, unable to analyze because of poor image quality

Direct photocoagulation was administered to 39 MAs meeting the treatment criteria. The anti-VEGF drug bevacizumab was injected into three eyes (cases 7, 8, and 12) at the time of photocoagulation.

**Fig. 1:**
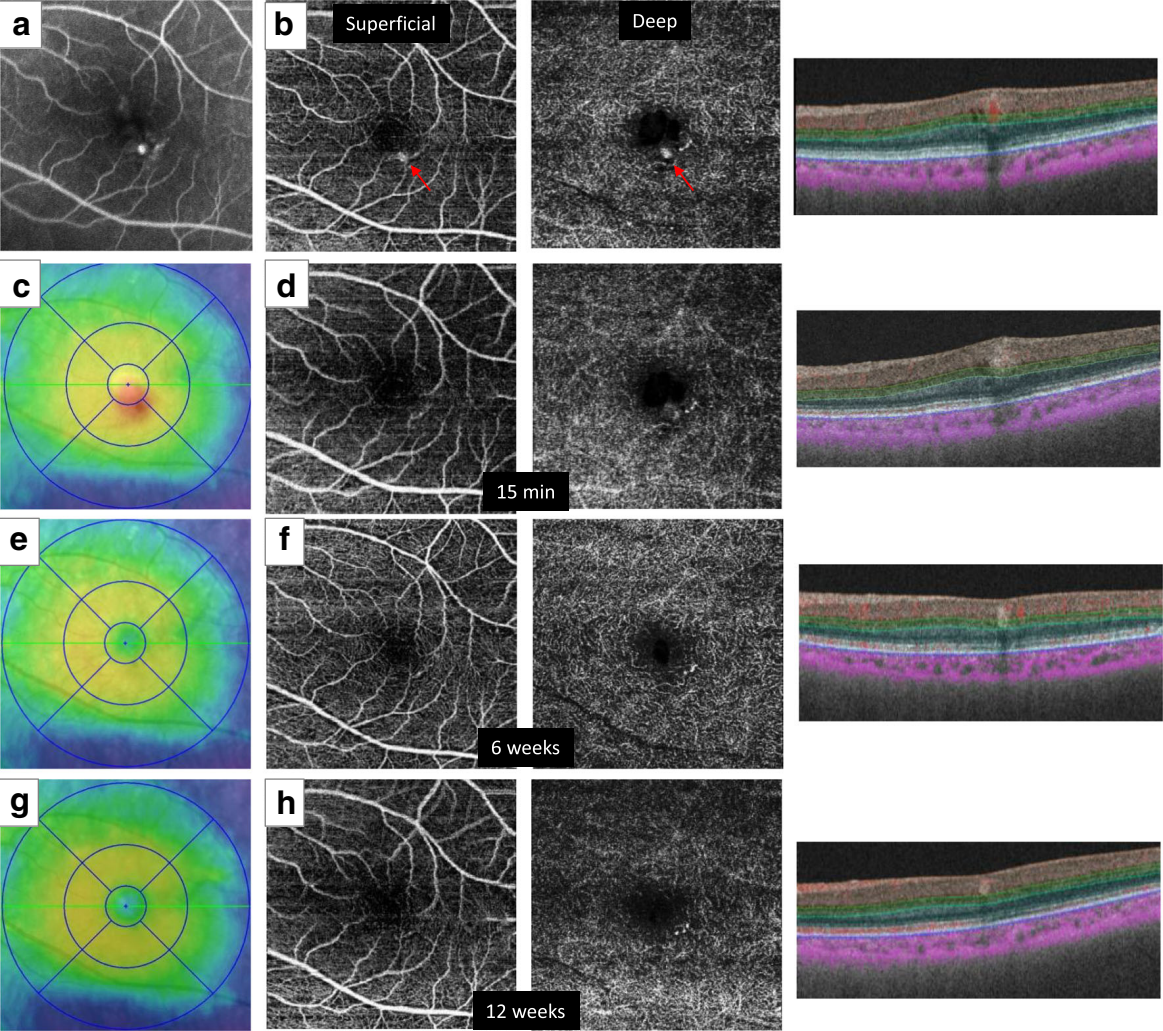
Images from the left eye of case 6. **a** Fluorescein angiography showed retinal microaneurysms (MAs). **b** Baseline enface and B scan optical coherence tomography angiography (OCTA) showed hyperreflective signals corresponding to MAs that were segmented into both the superficial and deep layers. One MA indicated with a red arrow was treated with photocoagulation. **c** Baseline OCT map showing a region of cystoid macular edema. **d** OCTA at 15 min showed no apparent signals within the treated MA region. **e** An OCT map at 6 weeks showed resolution of macular edema. **f** OCTA showed no signals within the treated MA. **g** An OCT map at 12 weeks showed no recurrence of edema. **h** OCTA also showed no recurrence of hyperreflective signals, and untreated small MAs were visible

**Fig. 2:**
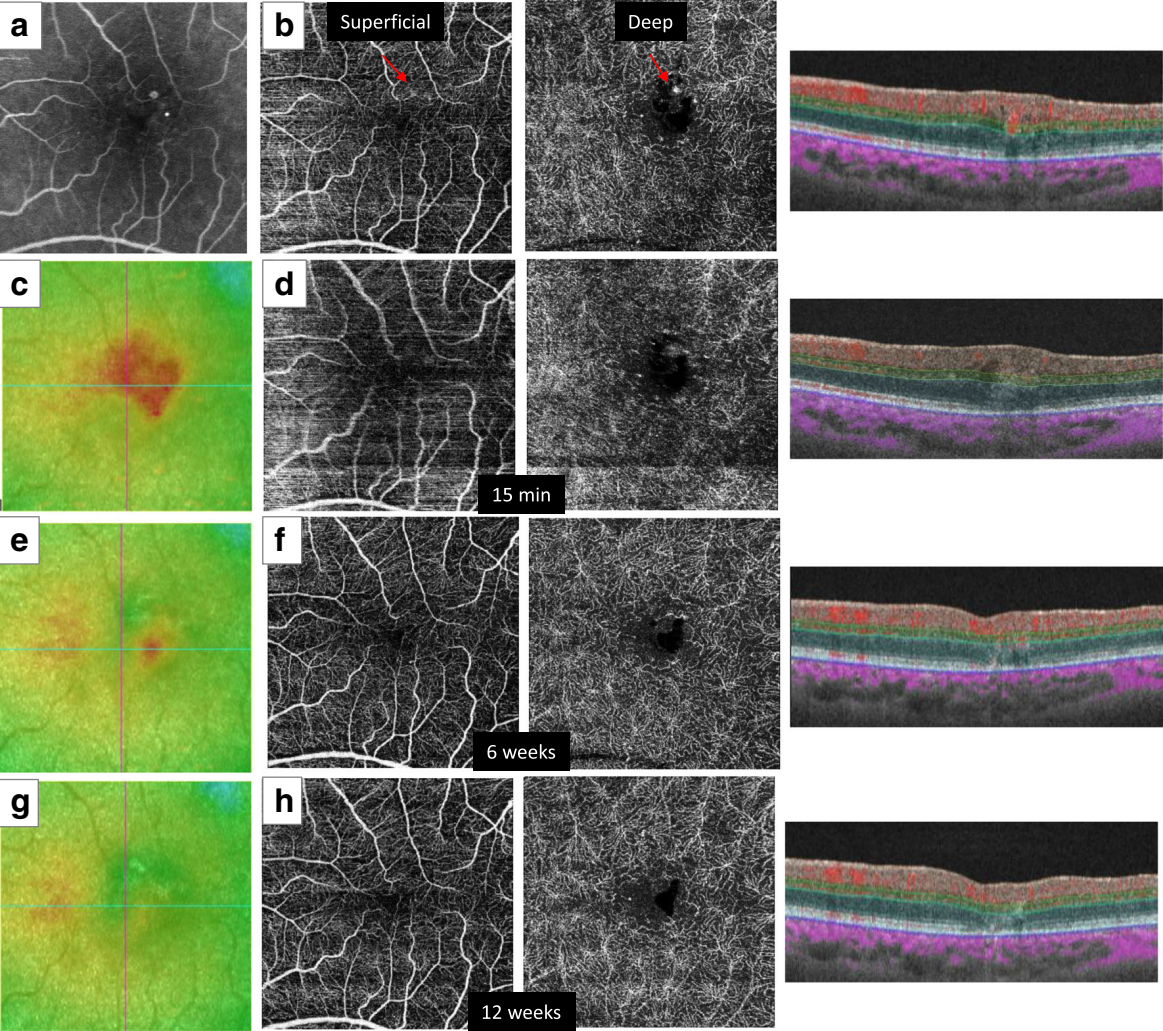
Images from the left eye of case 5. **a** Fluorescein angiography showed two retinal microaneurysms (MAs). **b** Baseline enface and B scan optical coherence tomography angiography (OCTA) showed hyperreflective signals corresponding to MAs that were segmented into both the superficial and deep layers. One MA indicated with a red arrow was treated with photocoagulation. **c** Baseline OCT map showing a region of cystoid macular edema. **d** OCTA at 15 min showed no hyperreflective signals within the treated MA region. **e** An OCT map at 6 weeks showed reduction of macular edema. **f** OCTA showed no apparent signals within the treated MA. **g** An OCT map at 12 weeks showed no retinal edema. **h** OCTA also showed no recurrence of hyperreflective signals and untreated small MAs were visible. B scan OCTA showed slight disruption of the outer retinal layer

The changes in the numbers of MAs depicted on OCTA after treatment are shown in Tables 2 and 3. At 15 min after photocoagulation, 17 of the MAs (45.9%), most of which were seen in both superficial and deep layers, could not be visualized on OCTA. Five MAs had disappeared by 6 weeks. Conversely, two MAs that were not visible at 15 min reappeared on 6 weeks. At this time, treated MAs were not visible in six (50%) eyes. Between 6 and 24 weeks, additional treatments including photocoagulation and anti-VEGF were performed in 6 eyes (Table 1). At 24 weeks, 13 of the 39 initially photocoagulated MAs (33.3%) were persistent. After 24 weeks, three eyes had new MAs and five eyes (cases 1, 2, 4, 9, and 12) had visible MAs on OCTA. Findings from representative cases are shown in Figs. 1 and 2.

**Table 3:** Changes in number of microaneurysms with photocoagulation

Case	Photocoagulated MAs (*n*)	Persistent MAs at 15 min (*n*)	Persistent MAs at 6 weeks (*n*)	Persistent MAs at 12 weeks (*n*)	Persistent MAs at 24 weeks (*n*)
1	2	1	1	1	1
2	1	0	1	1	1
3	2	2	2	2	0
4	16	7	5	5	5
5	1	0	0	0	0
6	1	0	0	0	0
7	2	N.A.	0	0	0
8	1	1	0	0	0
9	3	2	2	1	1
10	1	1	0	0	0
11	2	0	0	0	0
12	7	6	5	N.A.	5

* MAs, *microaneurysms*; N.A.*, unable to analyze because of poor image quality

The mean CRT of 340.08 ± 73.61 μm at 6 weeks was significantly less than that of 401.50 ± 52.29 μm at baseline (*p* = 0.0068). Except for three eyes with initially combined anti-VEGF therapy, the CRT in eyes with resolved MAs (cases 5, 6, 10, and 11) was significantly less than that in eyes with persistent MAs (cases 1, 2, 3, 4, and 9; *p* = 0.016). From 6 to 24 weeks, one additional anti-VEGF injection was administered in three eyes. Seven eyes had a foveal pit without a cyst and four eyes (cases 3, 4, 7, and 12) had a foveal pit with slight cystic changes, while the remaining eye (case 9) did not show a significant reduction in CRT. The mean BCVA was 0.11 ± 0.13 logMAR (range, − 0.08 to 0.30 logMAR) at 6 weeks, and 0.14 ± 0.14 logMAR (range, − 0.08 to 0.30 logMAR) at 24 weeks. These values were not significantly different from the baseline values (*p* = 0.12 and *p* = 0.48, respectively). At 24 weeks, no eyes developed retinal pigment epithelial atrophy, but two eyes showed a thinning of the outer retinal layer around the area of photocoagulation.

## Discussion

In this study, we could detect visible changes to MAs on OCTA before and after focal photocoagulation. Before treatment, MAs were seen preferentially in the deep retinal slab, as Matte et al. [15] reported previously, but relatively large MAs were found in both the superficial and deep slabs. Spaide et al. [16] also reported that some MAs in patients with diabetic retinopathy were seen in both the superficial and deep retinal slabs. However, as reported previously [7, 17, 18], not all MAs observed on FA or ICGA were delineated on OCTA. As we did not perform manual segmentation, some MAs could not be visualized because of errors in segmentation caused by retinal edema; repeated scans [19] and multiple image averaging [20] might increase the ability to detect MAs. Hard exudates were found to be the major cause of artifacts and were difficult to discriminate from MAs.

After photocoagulation, almost half of the treated MAs disappeared from OCTA within 15 min. All but two of the MAs that immediately disappeared were not apparent at 6 weeks and later. Furthermore, a few MAs that were visible just after photocoagulation were not visible at 6 weeks or later. Previously, in an analysis of FA, Sachdev et al. [21] observed that MA closure was only 0.67% at 2 weeks, but increased to 89.6% by 12 weeks. Using spectral-domain OCT, Lee et al. [22] and Yamada et al. [23] reported that MAs associated with diabetic maculopathy and that closed following focal laser photocoagulation showed uniform hyperreflectivity, finally disappearing over time. Immediately after photocoagulation, the lumen of MAs is changed and turbulent blood flow may occur within the treated MAs, making the MAs invisible on OCTA, as Nakao et al. reported. [24] Later, thrombus is formed, and the obstruction of blood flow within vessels can cause permanent closure of MAs. These time- and efficacy-dependent changes within the MAs after photocoagulation could cause a change in visibility on OCTA during the follow-up period.

Earlier disappearance of signals corresponding to the MAs on OCTA suggests the success of photocoagulation, although complete closure of MAs requires a long time, and in some cases, recanalization may occur during follow-up. In our study, the eyes in which MAs disappeared showed significant reductions in CRT, although 7 of 12 eyes with persistent MAs and CME required additional treatment over 24 weeks. Finally, all but one eye obtained a foveal pit. To avoid laser-induced damage, we selected the MAs to treat, and the results suggest that the resolution of CME depends on which MAs cause CME, and whether they are treatable with photocoagulation. Hirano et al. [25] reported that a combined focal/grid laser successfully reduced the number of anti-VEGF injections for diabetic macular edema. Therefore, we suggest that careful evaluation of OCTA images for MAs could reduce additional treatments with anti-VAGF and photocoagulation.

Regarding the BCVA, it had not improved significantly from baseline, although the CRT decreased significantly. A previous report showed that, in addition to the CRT, microvascular density, ellipsoid zone disruption, and the presence of disorganization in the retinal inner slabs might be predictive biomarkers of VA in Mac Tel type 1 [26]. We did not observe any retinal pigment epithelial atrophy during the post-treatment period, and only two eyes showed a thinning of the outer retinal layer around the area of photocoagulation; therefore, for photocoagulation to achieve its optimum effectiveness, a careful procedure targeting only selected MAs may be essential.

The limitations of this study are the small sample size and relatively short observation period. As mentioned earlier, the number of MAs could be inaccurate because of segmentation errors and artifacts. Furthermore, the use of combined anti-VEGF drugs depended on the physician.

In conclusion, the visualization of MAs on OCTA changed after direct photocoagulation. The results suggest that the success of photocoagulation can be monitored using noninvasive OCTA.

## Data Availability

Not applicable.
